# Biomarkers of metastatic disease in pheochromocytoma and paraganglioma

**DOI:** 10.1530/EC-26-0501

**Published:** 2026-08-25

**Authors:** Sara Donato, Aura D Herrera-Martínez, Verónica Alejandra Jacome-Gaibor, Ignacio Ruz-Caracuel, Rodrigo Toledo, Marta Araujo-Castro

**Affiliations:** ^1^ Endocrinology Department, Instituto Português de Oncologia de Lisboa Francisco Gentil, Lisbon, Portugal; ^2^ Facultad de Medicina de la Universidad de Alcalá, Alcalá de Henares, Spain; ^3^ Endocrinology Department, Hospital da Luz de Lisboa, Lisbon, Portugal; ^4^ Endocrinology & Nutrition Department, Hospital Universitario Reina Sofía, Córdoba, Spain; ^5^ Maimonides Institute for Biomedical Research of Córdoba, Córdoba, Spain; ^6^ Pathology Department, Hospital Universitario Ramón y Cajal, IRYCIS, Madrid, Spain; ^7^ CIBERONC, Madrid, Spain; ^8^ Vall d’Hebron Institute of Oncology (VHIO), Vall d’Hebron Barcelona Hospital Campus, Barcelona, Spain; ^9^ Endocrinology & Nutrition Department, Hospital Universitario Ramón y Cajal, Madrid, Spain; ^10^ Instituto de Investigación Biomédica Ramón y Cajal (IRYCIS), Madrid, Spain

**Keywords:** pheochromocytoma, paraganglioma, metastasis, biomarker, SDHB

## Abstract

Pheochromocytomas and paragangliomas (PPGLs) are rare neuroendocrine tumors with variable metastatic potential. While metastatic disease occurs in approximately 10–20% of cases, its prediction remains a major clinical challenge, as no histological system has been universally validated to reliably identify aggressive tumors at diagnosis. This review aims to provide a comprehensive and updated overview of current and emerging biomarkers of metastatic risk in PPGL, encompassing histopathological scoring systems, genetic and molecular markers, biochemical phenotyping, liquid biopsy approaches, and imaging-based biomarkers. Among established markers, germline *SDHB* mutation status, loss of SDHB expression by immunohistochemistry, elevated plasma 3-methoxytyramine, and histopathological scoring systems, such as GAPP and COPPS, represent the most clinically validated tools for risk stratification. Emerging biomarkers – including somatic alterations in *ATRX* and *TERT*, genomic instability indices, tumor immune microenvironment characterization, circulating tumor DNA, and oncometabolite quantification – show promise in refining prognostic assessment but require prospective validation before routine clinical implementation. Accurate risk stratification in PPGL demands a multiparametric and dynamic approach, integrating clinical, genetic, biochemical, and molecular parameters. Future progress will depend on large prospective international cohorts, standardized biomarker platforms, and biomarker-driven clinical trial designs to translate emerging molecular knowledge into improved patient outcomes.

## Introduction

The reported incidence of pheochromocytomas and paragangliomas (PPGLs) ranges from 0.04 to 0.95 cases per 100,000 persons per year (1). Over the past decades, the annual incidence has increased, largely due to the growing number of tumors detected incidentally during imaging studies performed for unrelated reasons (1, 2). These tumors affect both sexes and are most commonly diagnosed between the fourth and sixth decades of life, although they can occur across a wide age range (2).

From a molecular perspective, these tumors can be divided into three major transcriptomic clusters based on mRNA expression profiles (3). Cluster 1 tumors show the highest proportion of metastatic cases and are typically driven by somatic or germline mutations, affecting the cellular response to hypoxia. These alterations occur either in genes encoding enzymes involved in the tricarboxylic acid cycle or in genes that directly regulate the hypoxia-inducible factor pathway (4, 5). Cluster 2 tumors are characterized by mutations in genes regulating kinase signaling pathways. These tumors generally have lower metastatic potential. Finally, cluster 3 tumors are driven by *MAML3* gene fusions or *CSDE1* somatic mutations, which lead to aberrant activation of the Wnt signaling pathway. These tumors appear to carry an intermediate risk of metastatic disease (4, 5).

Historically, the terms benign and malignant pheochromocytoma were used in the 2004 WHO classification of endocrine tumors (6). However, these terms were abandoned in the 2017 WHO classification (7). By 2017, the WHO framework had shifted to a more unified concept: pheochromocytomas and paragangliomas were recognized as a single tumor family of chromaffin-cell neoplasms, with classification driven primarily by anatomic location, genetic background, and biologic behavior, rather than by a strict benign-versus-malignant dichotomy (7). The reason is that no histological system has been universally validated to reliably predict biological aggressiveness in these tumors (8). Consequently, all pheochromocytomas are now considered to have metastatic potential, and the term malignant has been replaced by metastatic because metastatic spread is the only definitive criterion of malignancy in these tumors. This change also avoids confusion between tumors that are locally invasive and those that truly metastasize to distant sites (7). The 2017 update also incorporated advances in tumor genetics and molecular biology, reflecting the recognition that inherited susceptibility syndromes and molecular subgroups are central to PPGL biology and clinical management.

In this review, we aim to update the current evidence on biomarkers of metastatic disease in pheochromocytoma and paraganglioma.

## Epidemiology, natural history, and current diagnosis criteria

The overall metastatic rate of PPGLs ranges approximately 10–20%; the risk is lower for pheochromocytomas and higher for paragangliomas, particularly extra-adrenal and *SDHB*-associated tumors (9, 10). Metastatic spread most commonly involves bones, regional or cervical lymph nodes, liver, and lungs, with head and neck paragangliomas showing a predominant predilection for cervical lymph node metastasis (9). When metastatic disease occurs, prognosis varies considerably. Reported 5- and 10-year survival rates for metastatic PPGL range broadly, reflecting the heterogeneity of the disease. Mortality rates vary, depending on factors such as primary tumor location, but overall mortality has been reported between 34 and 50% (11). Some studies also suggest a significant survival difference between patients with metastatic pheochromocytomas and those with metastatic paragangliomas after long-term follow-up (11).

Histopathology has an important adjunctive role in PPGL, but its ability to predict metastatic behavior is intrinsically limited, as no single histologic criterion can definitively establish malignancy (12). Several histopathological scoring systems have been developed to estimate metastatic potential and are discussed in detail elsewhere in this review.

The 2022 World Health Organization (WHO) Classification of Endocrine and Neuroendocrine Tumors no longer classifies PPGLs as benign or malignant because all tumors are considered to have metastatic potential. Instead, metastatic PPGL is currently defined by the presence of chromaffin tissue in non-physiological sites where chromaffin cells are normally absent, such as bone, liver, lungs, or distant lymph nodes (13, 14).

## Molecular pathogenesis of metastatic PPGL

**Figure 1:**
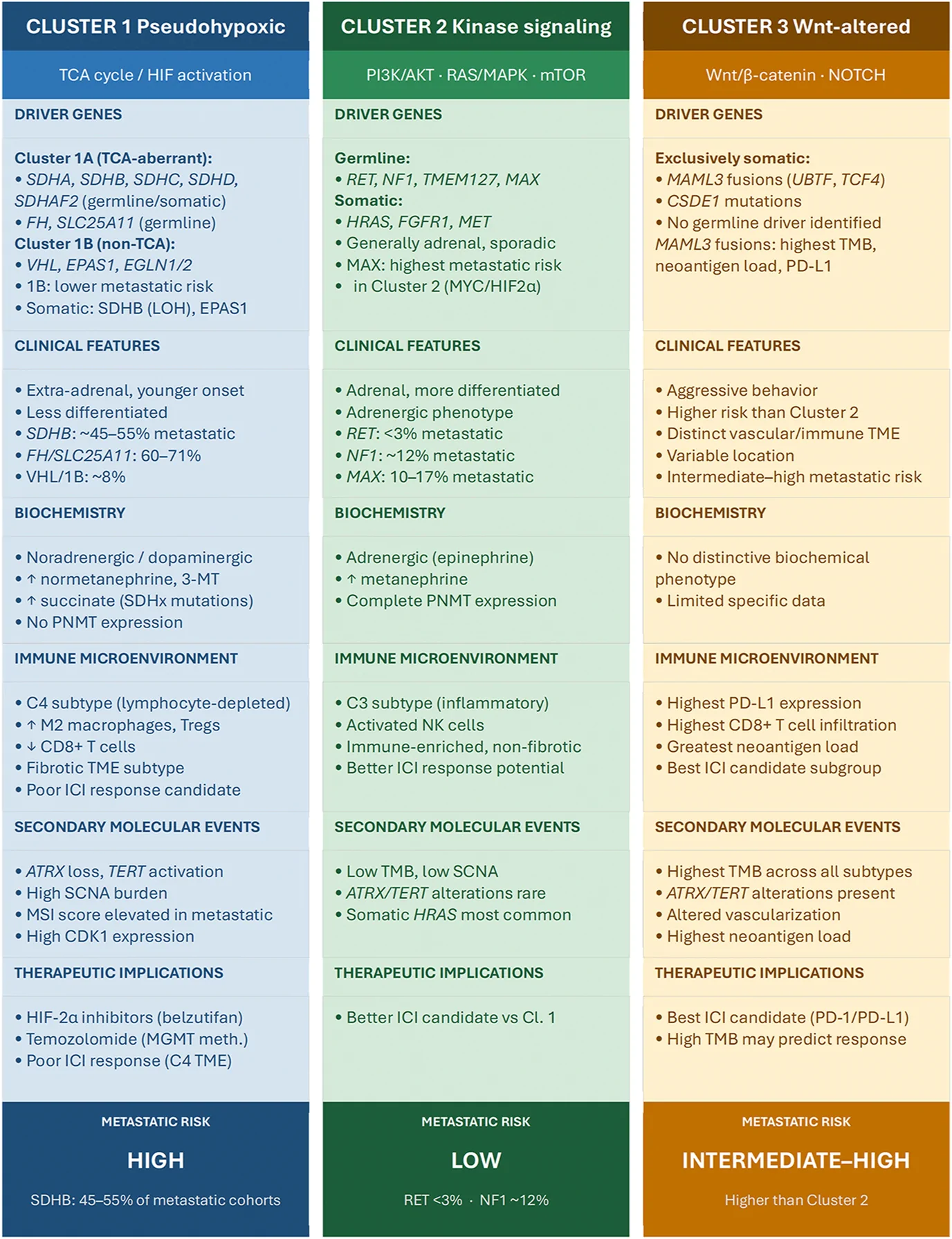
Molecular clusters in pheochromocytoma and paraganglioma. 3-MT: 3-methoxytyramine; ALT: alternative lengthening of telomeres; ATRX: alpha-thalassemia/mental retardation X-linked; CSDE1: cold shock domain-containing E1; EGLN1/2: egl-9 family hypoxia inducible factor 1/2; EPAS1: endothelial PAS domain protein 1 (HIF-2α); FH: fumarate hydratase; FGFR1: fibroblast growth factor receptor 1; HRAS: Harvey rat sarcoma viral oncogene; HIF: hypoxia-inducible factor; ICI: immune checkpoint inhibitor; LOH: loss of heterozygosity; MAML3: mastermind-like transcriptional coactivator 3; MAX: MYC-associated factor X; MGMT: O6-methylguanine-DNA methyltransferase; MET: MET proto-oncogene receptor tyrosine kinase; MSI: microsatellite instability; NF1: neurofibromin 1; NK: natural killer; PARP: poly(ADP-ribose) polymerase; PD-L1: programmed death-ligand 1; PNMT: phenylethanolamine N-methyltransferase; RET: rearranged during transfection proto-oncogene; SCNAs: somatic copy number alterations; SDHA/B/C/D/AF2: succinate dehydrogenase subunit A/B/C/D/assembly factor 2; SLC25A11: solute carrier family 25 member 11; TMB: tumor mutational burden; TME: tumor microenvironment; TMEM127: transmembrane protein 127; Tregs: T-regulatory cells; TERT: telomerase reverse transcriptase; TCF4: transcription factor 4; UBTF: upstream binding transcription factor; VHL: von Hippel–Lindau tumor suppressor.

Figure 1 presents a summary of the molecular clusters in pheochromocytoma and paraganglioma.

### Molecular clusters of PPGL

PPGLs are classified into three major molecular clusters according to the signaling pathways driving tumorigenesis. These molecular subtypes are associated with distinct biological mechanisms, biochemical phenotypes, and clinical behavior, providing an important framework for understanding disease pathogenesis (10, 13).

#### Cluster 1 (pseudohypoxic pathway)

Cluster 1 tumors are characterized by activation of hypoxia-inducible pathways despite normoxic conditions and are broadly divided into tricarboxylic acid (TCA)-cycle-related (cluster 1A) and non-TCA-related (cluster 1B) subtypes (13, 15, 16).

The TCA cycle aberrant tumors encompass germline or somatic mutations in enzymes involved in mitochondrial metabolism, including succinate dehydrogenase subunits *(SDHx–SDHA, SDHB, SDHC, SDHD, and SDHAF2*), fumarate hydratase (*FH*), malate dehydrogenase (*MDH2*), dihydrolipoamide S-succinyltransferase (*DLST*), isocitrate dehydrogenase (*IDH*), glutamic-oxaloacetic transaminase 2 (*GOT2*), and 2-oxoglutarate-malate carrier (*SLC25A11*) (10, 15, 16, 17). These defects disrupt oxidative metabolism, leading to the accumulation of oncometabolites, such as succinate and fumarate (13, 17). These metabolites competitively inhibit α-ketoglutarate-dependent dioxygenases, including ten-eleven translocation (TET) enzymes and prolyl hydroxylases (PHDs), resulting in stabilization of hypoxia-inducible factors (HIFs), genome-wide DNA hypermethylation, and activation of transcriptional programs involved in angiogenesis, metabolic reprogramming, proliferation, invasion, and metastatic progression (10, 13, 15, 16, 17, 18).

The non-TCA-related subgroup includes mutations affecting the pseudohypoxia response that are not primarily associated with the TCA cycle, such as germline mutations *in von Hippel–Lindau tumor suppressor* (*VHL*), *EGLN1/2* (*PHD1/2*), and somatic mutations in *VHL* and endothelial PAS domain protein 1 (*EPAS1*), encoding HIF2α (15, 16, 17). These alterations also lead to HIF stabilization and activation of pseudohypoxia signaling but are generally associated with less pronounced epigenetic dysregulation than TCA-deficient tumors (15, 17).

Overall, cluster 1 tumors are typically less differentiated, often extra-adrenal, and lack the complete catecholamine synthesis pathway, resulting in elevated dopamine and norepinephrine, along with their metabolites, 3-methoxytyramine and normetanephrine (14). This cluster’s tumors carry the greatest metastatic potential among the three molecular subtypes, although the risk varies substantially according to the specific underlying genetic alteration, as discussed in the Genetic Biomarkers section (10, 15, 16, 17).

#### Cluster 2 (tyrosine kinase-linked signaling pathway)

Cluster 2 includes tumors harboring pathogenic variants affecting kinase signaling pathways, including *RET* proto-oncogene (*RET*), neurofibromin 1 (*NF1*), transmembrane protein 127 (*TMEM127*), and MYC-associated factor X (*MAX*), as well as somatic mutations in Harvey rat sarcoma viral oncoprotein (*HRAS*), fibroblast growth factor receptor 1 (*FGFR1*), and MET proto-oncogene receptor tyrosine kinase (*MET*) (10, 16, 17). These alterations activate cascades such as PI3K/AKT, RAS/RAF/ERK, and mTORC1, promoting sustained cell growth, survival, proliferation, and angiogenesis (10, 17).

Compared with cluster 1, these tumors are generally more differentiated, adrenal in origin, and exhibit an adrenergic biochemical phenotype, with preserved epinephrine synthesis (10, 15, 16, 17, 18). Although most cluster 2 tumors demonstrate relatively low metastatic potential, clinically relevant differences exist between individual genes and are addressed in the Genetic Biomarkers section.

#### Cluster 3 (Wnt signaling-related cluster)

Cluster 3 is characterized by activation of Wnt/β-catenin signaling through exclusively somatic alterations, most commonly MAML3 fusion events and CSDE1 mutations (10, 15, 16, 17, 18). These alterations activate transcriptional programs involved in angiogenesis, proliferation, invasion, metastatic dissemination, and remodeling of the tumor microenvironment, contributing to an aggressive biological phenotype (15, 17, 19).

Although relatively uncommon, tumors belonging to this molecular cluster exhibit aggressive behavior and contribute disproportionately to metastatic disease (15, 16).

### Tumor immune microenvironment and immune phenotype subtypes

Beyond the transcriptomic clusters defined by driver gene alterations, an emerging and complementary dimension of PPGL biology is the characterization of the tumor microenvironment (TME). PPGLs have historically been regarded as immunologically ‘cold’ tumors, characterized by low tumor mutational burden (TMB), limited tumor-infiltrating lymphocytes, and a microenvironment largely devoid of effector immune cells (20, 21). However, recent large-scale genomic and immunogenomic studies have challenged this view and revealed a heterogeneous immune landscape that correlates with molecular subtype and metastatic behavior (21).

Calsina *et al.* applied the immune subtype classification proposed by Thorsson *et al.* – based on immunogenomic analysis of more than 10,000 tumors from The Cancer Genome Atlas – to a large PPGL dataset, identifying three relevant immune subtypes: C3 (inflammatory), C4 (lymphocyte depleted), and C5 (immunologically quiet) (21, 22). Of these, C3 was predominantly enriched in non-metastatic tumors (52.3%), whereas C4 showed significantly higher prevalence among primary metastatic tumors and metastases (50.9 and 71.4%, respectively). Patients with tumors classified as C4 also exhibited shorter time to progression compared with those with tumors classified as C3 (21). The C4 immune subtype can be viewed as permissive of disease aggression: it is characterized by the absence of effector cytotoxic CD8+ T cells, abundance of M2-like macrophages – known for their anti-inflammatory and pro-tumoral role – and enrichment of T-regulatory cells (Tregs), collectively establishing an immunosuppressive ecosystem (20, 21). This pattern was further confirmed by immune cell deconvolution analysis, which demonstrated significantly lower proportions of activated CD4+ and CD8+ T cells, alongside higher numbers of M2-like macrophages and resting NK cells in metastatic primary tumors compared with non-metastatic ones (21).

Different immune phenotypes also correlate with the underlying genomic subtype. The pseudohypoxic cluster (cluster 1) is predominantly enriched in the C4 immune subtype and in the fibrotic TME subtype (characterized by high vascularity, TGF-β-driven immunosuppression, and low lymphocytic infiltration), which has been associated with poor response to immunotherapy across multiple cancer types (20, 21). This immunosuppressive phenotype in pseudohypoxic tumors appears to be mechanistically driven by HIF2α activation, which upregulates lactate dehydrogenase A (LDHA), a known mediator of immunosuppression through lactic acid production, thereby impairing cytotoxic T cell function and promoting M1-to-M2 macrophage polarization (20). In addition, excessive catecholamine levels, characteristic of metastatic PPGLs, may further contribute to immune suppression by diminishing T cell and NK cell activation through β-adrenoceptor signaling (20).

In contrast, the kinase signaling cluster (cluster 2) is more represented in immune cluster 3 (characterized by activated NK cells) and in the immune-enriched, non-fibrotic TME subtype, which is associated with better prognosis and higher response rates to immunotherapy (20, 21). Notably, MAML3-fused tumors display a distinct immune phenotype, with the highest PD-L1 expression, the greatest neoantigen load, and the highest CD8+ T cell infiltration among all PPGL molecular subtypes (21). This suggests that a subset of cluster 3 tumors, particularly those driven by *MAML3* fusions, may represent the most immunotherapeutically tractable PPGL subgroup, potentially amenable to PD-1/PD-L1 checkpoint inhibition (20, 21).

Complementary to this immune subtyping approach, Ghosal *et al.* independently identified five PPGL-specific immune clusters based on immune cell infiltration profiles (23).

Immune cluster 3, enriched in NK cell-related chemokines (CCL3 and CCL4), was associated with non-metastatic cases and better prognosis, while immune cluster 4 – defined by macrophage and Treg predominance – was composed predominantly of metastatic tumors (20, 23). These findings converge with those of Calsina *et al.* and collectively support the concept that an immunosuppressive TME, dominated by M2 macrophages and Tregs with depletion of cytotoxic effectors, is a hallmark of aggressive PPGL behavior, particularly in the pseudohypoxic subgroup (21).

These data highlight the potential clinical utility of immune phenotyping as an additional layer of PPGL risk stratification, complementary to genomic subtyping. Understanding the immune contexture of individual tumors may not only refine prognostic classification but also guide the selection of immunotherapeutic strategies, particularly as clinical trials exploring checkpoint inhibitors and combination approaches in metastatic PPGL continue to emerge.

## Genetic biomarkers

In Table 1, we present a summary of all genetic biomarkers of metastatic risk in PPGL discussed in this paper.

**Table 1:** Summary of genetic biomarkers of metastatic risk in PPGL.

Gene/alteration	Type	Cluster	Metastatic risk	Key features	Refs
**Germline SDHx mutations**					
*SDHB*	Germline	1A	∼45–55% of all metastatic PPGLs; up to 80% in pediatric cases	Extra-adrenal location; noradrenergic phenotype; younger age at presentation; TERT/ATRX as secondary drivers	(9, 19, 20, 28)
*SDHA*	Germline	1A	∼16%	PPGL5 syndrome; succinate accumulation; pseudohypoxia	(9, 21)
*SDHC*	Germline	1A	∼23%	PPGL3 syndrome; predominantly head and neck PGLs	(9, 21)
*SDHD*	Germline	1A	∼6–8%	PPGL1 syndrome; multiple tumors; head and neck PGLs; maternal imprinting	(9, 21)
*SDHAF2*	Germline	1A	No metastatic events reported	Head and neck PGLs	(21)
**Other germline mutations – cluster 1**					
*FH*	Germline	1A	∼60%	Rare; fumarate accumulation; similar mechanism to SDHB; highly aggressive	(9, 20, 21)
*SLC25A11*	Germline	1A	∼71%	Novel susceptibility gene; SDHx-like phenotype; highly aggressive	(21, 28)
*VHL*	Germline	1B	∼8%	Pseudohypoxia via HIF accumulation; predominantly benign behavior despite pathway activation	(9, 20, 21)
*EGLN1*	Germline	1B	Elevated (exact rate uncertain)	Pseudohypoxia; rare	(9, 28)
*KIF1B*	Germline	1B	Elevated	Rare; associated with increased metastatic risk	(9, 28)
**Other germline mutations – cluster 2**					
*RET*	Germline	2	<3%	Adrenal; adrenergic phenotype; MEN2; PI3K/AKT activation	(9, 20, 24)
*NF1*	Germline	2	∼12%	Adrenal; RAS/MAPK activation; typically sporadic presentation	(9, 20, 21)
*TMEM127*	Germline	2	∼2%	Predominantly benign; mTORC1 signaling	(9, 20, 24)
*MAX*	Germline	2	∼10–17%	Disproportionately high risk for cluster 2; MYC/HIF2α interaction	(9, 20, 24)
**Somatic alterations**					
Somatic *SDHB*	Somatic	1A	Similar to germline (data limited)	Predominantly via LOH at 1p36; same downstream cascade as germline; SDHB IHC as surrogate	(5, 9, 20, 31)
*EPAS1*	Somatic	1B	∼30%	5–8% of all PPGLs; extra-adrenal; noradrenergic; HIF-2α gain-of-function; belzutifan target	(9, 20, 21)
*TERT*	Somatic	1A/Wnt	Independent adverse prognostic factor	Telomerase activation; structural rearrangements > promoter mutations; enriched in SDHB/FH and Wnt-altered tumors	(19, 20, 28)
*ATRX*	Somatic	1A/Wnt	Independent risk factor; worse OS and MFS	ALT pathway; mutually exclusive with TERT; SDHB/FH-enriched; detectable by IHC	(5, 19, 20, 28, 29)
Somatic *MAML3* fusion/*CSDE1*	Somatic	3 (Wnt)	Higher than cluster 2; aggressive behavior	Highest TMB, neoantigen load, PD-L1 expression; best immunotherapy candidate	(5, 20, 26)

ATRX, alpha-thalassemia/mental retardation X-linked; CSDE1, cold shock domain-containing E1; EGLN1, egl-9 family hypoxia inducible factor 1; EPAS1, endothelial PAS domain protein 1 (HIF-2α); FH, fumarate hydratase; HIF, hypoxia-inducible factor; IHC, immunohistochemistry; KIF1B, kinesin family member 1B; LOH, loss of heterozygosity; MAML3, mastermind-like transcriptional coactivator 3; MAX, MYC-associated factor X; MFS, metastasis-free survival; NF1, neurofibromin 1; OS, overall survival; PARP, poly(ADP-ribose) polymerase; PD-L1, programmed death-ligand 1; PPGL, pheochromocytoma/paraganglioma; RET, rearranged during transfection proto-oncogene; SDHA/B/C/D/AF2, succinate dehydrogenase subunit A/B/C/D/assembly factor 2; SLC25A11, solute carrier family 25 member 11; TCF4, transcription factor 4; TERT, telomerase reverse transcriptase; TMB, tumor mutational burden; TMEM127, transmembrane protein 127; UBTF, upstream binding transcription factor; VHL, von Hippel–Lindau tumor suppressor.

### Germline mutations

While molecular clustering provides the biological framework for PPGL pathogenesis, metastatic risk ultimately depends on the specific genetic alterations present within each molecular subtype (8, 10). Although tumors belonging to the same molecular cluster share common pathogenic mechanisms, substantial differences in clinical behavior exist between individual genes. Therefore, characterization of germline and somatic biomarkers provides prognostic information beyond molecular clustering and is essential for individualized risk stratification, surveillance strategies, and therapeutic decision-making.

#### SDHB

Germline *SDHB* mutation is associated with the higher metastatic risk in patients with PPGL, which appears in about half of the *SDHB* mutated cases (10, 13, 24). Due to this high risk, *SDHB* patients require close monitoring, and when surgery is performed, no residual tissue should be left behind (15).

A recent genomic study of *SDHB*-mutated PPGLs identified *TERT* promoter mutations and *ATRX* loss as the most frequent secondary driver events, both strongly associated with metastatic disease and mutually exclusive, suggesting overlapping roles in telomere maintenance and cell cycle regulation (25).

#### Other SDHx genes

Pathogenic germline mutations in other *SDH* subunits are also associated with elevated metastatic risk, although with variable penetrance (10, 15, 16). *SDHA* mutations (PPGL5 syndrome) are associated with a metastatic rate of approximately 16%, while *SDHC* mutations (PPGL3 syndrome), predominantly associated with head and neck PGLs, carry a metastatic rate of approximately 23% (10, 16). *SDHAF2* mutations have not been associated with metastatic events to date (16). As further detailed in the following, *SDHD* mutations (PPGL1 syndrome) carry a comparatively lower metastatic rate of 6–8% (10, 16).

#### Other mutations

Among non-SDHx variants, *FH, SLC25A11, MAX, KIF1B*, and *EGLN1* germline mutations have all been significantly associated with increased metastatic risk compared with patients with no identified variant (10, 24). Of these, *FH* and *SLC25A11* carry particularly high metastatic potential (∼60 and 71%, respectively). In contrast, kinase-signaling variants (*RET, NF1, TMEM127*) are generally associated with lower metastatic risk, although *MAX* represents a notable exception within this cluster (10, 15, 16, 18, 24, 26, 27).

These observations highlight that molecular cluster assignment alone is insufficient to estimate metastatic risk and should be complemented by gene-specific risk assessment.

#### Penetrance and genotype–phenotype correlations

Genotype–phenotype correlations are well established in PPGL and represent one of the most clinically useful frameworks for metastatic risk stratification. However, penetrance varies considerably, even among carriers of the same pathogenic variant, indicating that genotype alone is insufficient to accurately predict individual outcomes (8, 10, 15, 25).

Beyond individual genes, molecular subtype also provides important prognostic information. Tumors belonging to the pseudohypoxic cluster exhibit significantly higher metastatic potential than kinase-signaling tumors, with pseudohypoxic genotypes conferring approximately six-fold greater odds of metastasis in multivariable analyses (28). Nevertheless, substantial heterogeneity exists within each molecular cluster. For example, although most cluster 1 tumors are associated with increased metastatic risk, VHL-related PPGLs generally follow a more indolent clinical course, whereas FH and SDHB alterations are associated with particularly aggressive disease. Conversely, although cluster 2 tumors usually display low metastatic potential, MAX mutations represent an important exception (10, 15, 16, 18, 24, 26, 27, 28).

Additional molecular events further modify metastatic risk independently of the underlying germline genotype. In particular, ATRX loss, TERT activation, high microsatellite instability scores, and increased CDK1 expression have emerged as important secondary drivers of metastatic progression, especially in SDHB-associated tumors (10, 13, 15, 21, 24, 25). These observations further support the concept that metastatic progression results from the interaction between the initiating genetic alteration and subsequent molecular events acquired during tumor evolution.

Biochemical phenotype also contributes to risk stratification. Dopamine-secreting tumors and elevated plasma 3-methoxytyramine, features predominantly observed in pseudohypoxic PPGLs, are independently associated with metastatic disease, with dopamine secretion conferring an odds ratio of 6.39 for metastasis (15, 28).

Collectively, these findings support an integrated approach, combining molecular cluster, genotype, biochemical phenotype, tumor size, anatomical location, and secondary somatic alterations for individualized metastatic risk assessment and clinical management (15, 19, 28).

### Somatic alterations

#### Pseudohypoxia-related alterations

##### Somatic SDHB

True somatic *SDHB* pathogenic variants isolated to tumor tissue are uncommon in PPGL. Unlike germline *SDHB* mutations, somatic point mutations have rarely been identified in apparently sporadic cases, and biallelic inactivation through loss of heterozygosity (LOH) at the 1p36 locus appears to be a more prevalent mechanism of somatic *SDHB* inactivation than point mutation per se (10, 29). Regardless of the underlying mechanism, somatic *SDHB* loss triggers the same downstream cascade as germline mutations – succinate accumulation, pseudohypoxia, and genome-wide hypermethylation – suggesting a similarly aggressive tumor biology (5, 10, 15).

From a practical standpoint, SDHB immunohistochemistry captures biallelic *SDH* complex inactivation irrespective of its cause, whether germline mutation, somatic mutation, LOH, or epigenetic silencing, and therefore, serves as the most clinically applicable surrogate biomarker in tumors, lacking an identifiable germline variant (5, 13, 30, 31). Dedicated studies characterizing the frequency and metastatic risk of purely somatic *SDHB* inactivation independently from germline cases remain scarce, and future whole-genome sequencing approaches will be essential to fully define this subgroup (15).

##### EPAS1

Somatic gain-of-function mutations in *EPAS1*, encoding HIF-2α, account for approximately 5–8% of all PPGLs and are classified within cluster 1B (10, 15, 16, 32). These tumors predominantly arise from the adrenal medulla and abdominal sympathetic ganglia, display a noradrenergic biochemical phenotype, and carry an estimated metastatic risk of approximately 30% (10, 15, 16). Notably, somatic *EPAS1* mutations are frequently mosaic, present in only a subset of tumor cells, which may result in multifocal or recurrent disease and can complicate mutation detection by using standard sequencing approaches (33). From a therapeutic standpoint, the identification of a somatic *EPAS1* variant may improve access to targeted therapies as HIF-2α inhibitors, such as belzutifan, are currently under clinical investigation in patients with advanced PPGL (15, 16).

This illustrates how identification of specific somatic alterations may have both prognostic and therapeutic implications, reinforcing the importance of integrating molecular profiling into the management of advanced PPGL.

#### Telomere maintenance mechanisms

Normal somatic cells undergo replicative senescence after a finite number of divisions due to progressive telomere shortening. Tumor cells overcome this limitation by maintaining telomere length through two main mechanisms: telomerase activation, typically via *TERT,* and alternative lengthening of telomeres (ALT), frequently associated with loss of function alterations in alpha-thalassemia/mental retardation X-linked (*ATRX*) (15, 19).

Telomerase activation represents an independent adverse prognostic factor for overall survival (15, 20). Similarly, *ATRX* mutations are independent risk factors for metastatic PPGL and are associated with worse metastasis-free and overall survival (15, 24, 25). These alterations are linked to activation of the ALT pathway and are predominantly observed in *SDHB*/*FH*-mutated and Wnt-altered metastatic tumors (15, 21). These alterations are associated with increased genomic instability, including higher tumor mutation burden, microsatellite instability (MSI), and somatic copy number alterations (SCNAs), as well as increased proliferative indices, such as MKI67/Ki-67 expression and larger tumor size (13, 15, 21).

Epigenetic activation of *TERT,* particularly through promoter methylation, has also been identified as a valuable marker for risk stratification and an independent predictor of metastatic disease in PPGL (15, 24, 25). Somatic telomere maintenance by *TERT* promoter mutation is an independent risk factor for metastatic PPGL (15, 24). In contrast to many other cancers, where *TERT* activation commonly occurs through promoter mutations, telomerase activation in metastatic PPGL is more frequently driven by structural rearrangements that upregulate *TERT* expression and promote telomere elongation (15).

Collectively, these findings support the concept that dysregulation of telomere maintenance, enabling cellular immortalization, is a key determinant of poor prognosis in PPGL (21).

#### Genomic instability and chromatin remodeling

##### Somatic copy number alterations

SCNAs are a risk factor for aggressive behavior in PPGL, with no major differences observed across genomic subtypes (15, 19, 21). Increased SCNA burden is associated with shorter time to progression and is frequently observed in *ATRX-* and *TERT*-altered tumors (17, 21).

Recurrent chromosomal alterations include gains of 1q, 5p, and 5q, as well as deletions of 3q (21). Notably, whole chromosome 5 gain has been associated with shorter time to progression, consistent with the localization of the *TERT* locus at 5p15.33 (21).

In addition to structural genomic alterations, transcriptomic analyses have identified gene expression programs associated with metastatic PPGL (21). Upregulated pathways in metastatic tumors include those related to cell cycle progression, DNA repair, p53 signaling, extracellular matrix organization, protein translation, and proteasome activity, reflecting increased proliferative and metabolic demands (21). In contrast, downregulated pathways involve ciliogenesis, ion transport, Rho GTPase signaling, cell adhesion, neuronal differentiation, and immune response, suggesting loss of differentiation and immune evasion (21). Notably, cilia loss and Rho pathway deregulation have previously been implicated in PPGL tumorigenesis, supporting their role in malignant transformation (21).

Among candidate gene expression markers, *CDK1* has shown a particularly strong association with metastatic risk (21). In combined cohort analyses, high CDK1 expression was significantly associated with increased risk of metastasis (odds ratio (OR) ≈ 9.0) and shorter time to progression (hazard ratio (HR) ≈ 4.1), independent of genomic subtype (21). These findings highlight the potential role of cell cycle-related transcriptional programs in driving aggressive PPGL behavior, although further validation is required before clinical implementation.

PPGLs are characterized by one of the lowest median TMB and neoantigen loads among all cancers cataloged in The Cancer Genome Atlas (TCGA), a feature that likely contributes to the lymphocyte-depleted microenvironment, a characteristic of these tumors (21). Nevertheless, TMB is not uniformly low across all PPGL subgroups. Calsina *et al.* demonstrated that primary metastatic PPGLs and metastases harbor significantly higher TMB than non-metastatic primary tumors and that increased TMB and MSI scores were both associated with shorter time to progression (21). Across genomic subtypes, the Wnt-altered cluster – and particularly *MAML3*-fused tumors – consistently showed the highest TMB and neoantigen load, regardless of metastatic behavior (21). This molecular subtype, therefore, combines an aggressive clinical phenotype with the highest tumor mutational burden, neoantigen load, and PD-L1 expression among PPGL molecular subtypes, highlighting its potential relevance for future biomarker-driven therapeutic strategies. However, TMB alone appears insufficient as a predictive marker in PPGL: available evidence indicates that TMB does not reliably correlate with cytotoxic immune cell infiltration or immunophenotype and should, therefore, be interpreted alongside other factors, such as neoantigen clonality and the broader tumor immune microenvironment, rather than being used in isolation to guide immunotherapy decisions (20, 21).

Collectively, these findings underscore that metastatic risk in PPGL cannot be captured by a single molecular marker. Emerging tools – including TMB, MSI, genomic instability indices, and oncometabolite signatures – show promise in refining risk stratification, but most remain investigational. Future progress will likely depend on integrated models combining clinical, pathological, genetic, and metabolic parameters to enable accurate prognostication and personalized management (15, 21).

## Histopathological and immunohistochemical biomarkers

As we previously mentioned, the term ‘malignant’ pheochromocytoma and paraganglioma was abandoned in the 2017 WHO classification, which recognized that these tumors have variable metastatic potential. To predict this metastatic potential, histopathological scoring systems and key immunohistochemical biomarkers have been developed (14).

### Scoring systems

Four scoring systems have been proposed: PASS, GAPP, M-GAPP, and COPPS (Table 2) (30, 31, 34, 35).

**Table 2:** Comparative overview of the four main histopathological scoring systems.

	PASS	GAPP	M-GAPP	COPPS
Score range	0–20	0–10	0–10	0–10
Number of parameters	12	6	6	5
Immunohistochemical parameters included	None	Ki-67	Ki-67, SDHB	SDHB, S100
Key features	High negative predictive value	High negative predictive value	Improved accuracy related to GAPP	High positive predictive value

PASS, Pheochromocytoma of the Adrenal Gland Scaled Score; GAPP, Grading System for Adrenal Pheochromocytoma and Paraganglioma; M-GAPP, Modified Grading System for Adrenal Pheochromocytoma and Paraganglioma; COPPS, Composite Pheochromocytoma/Paraganglioma Prognostic Score.

#### PASS (Pheochromocytoma of the Adrenal Gland Scaled Score)

Developed by Thompson in 2002, PASS is a weighted scale of 12 histological parameters (such as vascular invasion, necrosis, and typical/atypical mitoses) designed exclusively for adrenal tumors. The score ranges from 0 to 20. A PASS score < 4 has a high negative predictive value, reliably identifying tumors with low risk of aggressive behavior. One advantage of this system is that it can be applied using only hematoxylin–eosin sections of the primary tumor (34). As a limitation, it shows high interobserver and intraobserver variability, even among expert pathologists, largely due to the subjective nature of criteria such as marked nuclear pleomorphism or atypical mitoses (36). In addition, its applicability is limited to pheochromocytomas.

#### GAPP (Grading System for Adrenal Pheochromocytoma and Paraganglioma)

Introduced by Kimura in 2014, GAPP classifies tumors as well, moderately and poorly differentiated based on histological pattern, cellularity, necrosis, invasion, Ki-67 index, and the type of catecholamine secreted (35). The total score ranges from 0 to 10. Unlike PASS, GAPP can be applied to both pheochromocytomas as well as sympathetic paragangliomas and has shown improved reproducibility and interobserver agreement (36). It correlates significantly with 5-year survival and time to metastasis. However, completion of the scoring system requires clinical biochemical data (catecholamine phenotype), which can be a barrier if such information is unavailable.

#### M-GAPP (Modified Grading System for Adrenal Pheochromocytoma and Paraganglioma)

Proposed by Koh *et al.* in 2017, M-GAPP is a modified version of the GAPP system, designed to improve diagnostic accuracy. It retains six parameters from the original model but replaces tumor cellularity with loss of SDHB protein expression assessed by immunohistochemistry. Similar to GAPP, the total score ranges from 0 to 10. This modification aims to incorporate the well-established association between SDHB deficiency and increased metastatic risk in pheochromocytomas and paragangliomas, thereby enhancing the system’s ability to predict aggressive behavior. However, its application requires SDHB immunohistochemical evaluation, which may not always be routinely available in all pathology laboratories (30).

#### COPPS (Composite Pheochromocytoma/Paraganglioma Prognostic Score)

Proposed by Pierre *et al.* in 2019, COPPS is a risk-stratification system that integrates three clinicopathological parameters – tumor size > 7 cm, focal or confluent necrosis, and vascular invasion – with two immunohistochemical markers, loss of S100 and loss of SDHB expression. The total score ranges from 0 to 10. This composite approach has demonstrated high diagnostic performance for predicting metastatic risk and progression-free survival. In addition, COPPS shows excellent interobserver reproducibility among pathologists (weighted kappa coefficient 0.863, in the original publication), which helps reduce the subjectivity associated with earlier histopathological scoring systems (31).

Among the proposed scoring systems, PASS and GAPP are the most extensively validated in the literature. Validation studies indicate that their performance is characterized by a relatively high negative predictive value, allowing reliable identification of tumors with a low likelihood of aggressive behavior. In contrast, COPPS was designed to achieve a higher positive predictive value for predicting metastatic potential. Therefore, these systems may be considered complementary tools for risk stratification (36, 37).

### Immunohistochemical biomarkers

Ki-67 is a key marker of cellular proliferation in the risk stratification of neuroendocrine tumors. A high Ki-67 index is positively associated with metastatic disease and lower recurrence-free survival (35, 38). It is directly incorporated into the GAPP score: 0 points for <1%, 1 point for 1–3%, and 2 points for >3%. Although it is a strong predictor, tumors with low Ki-67 (<1%) have also been observed to metastasize, underscoring that it should not be used as a sole indicator of malignancy.

Loss of granular cytoplasmic SDHB staining indicates the presence of a germline or somatic mutation in any of the *SDHx* complex genes (*SDHA*, *SDHAF2*, *SDHB*, *SDHC*, or *SDHD*) (38, 39). Mutations in *SDHB* are an independent and robust risk factor for metastatic progression and reduced disease-specific survival, as previously discussed.

S100 protein is commonly used to highlight sustentacular cells surrounding the chief cell nests (‘zellballen’ structures). A reduction or loss of sustentacular cells has been associated with more aggressive biological behavior and an increased risk of metastasis (31, 38). For this reason, S100 expression is included as a parameter in the COPPS scoring system (31). In addition, S100 immunohistochemistry may help distinguish multifocal primary tumors from metastatic disease, as metastases typically lack a well-developed sustentacular framework compared with primary tumors (14).

Loss of nuclear ATRX expression by immunohistochemistry reflects underlying somatic mutations in the *ATRX* gene and is closely associated with the alternative lengthening of telomeres pathway, a mechanism of cellular immortalization. ATRX deficiency has been significantly associated with metastatic disease, shorter metastasis-free survival, and reduced overall survival, particularly in tumors with *SDHB* or *FH* mutations (5, 21, 25).

**Figure 2:**
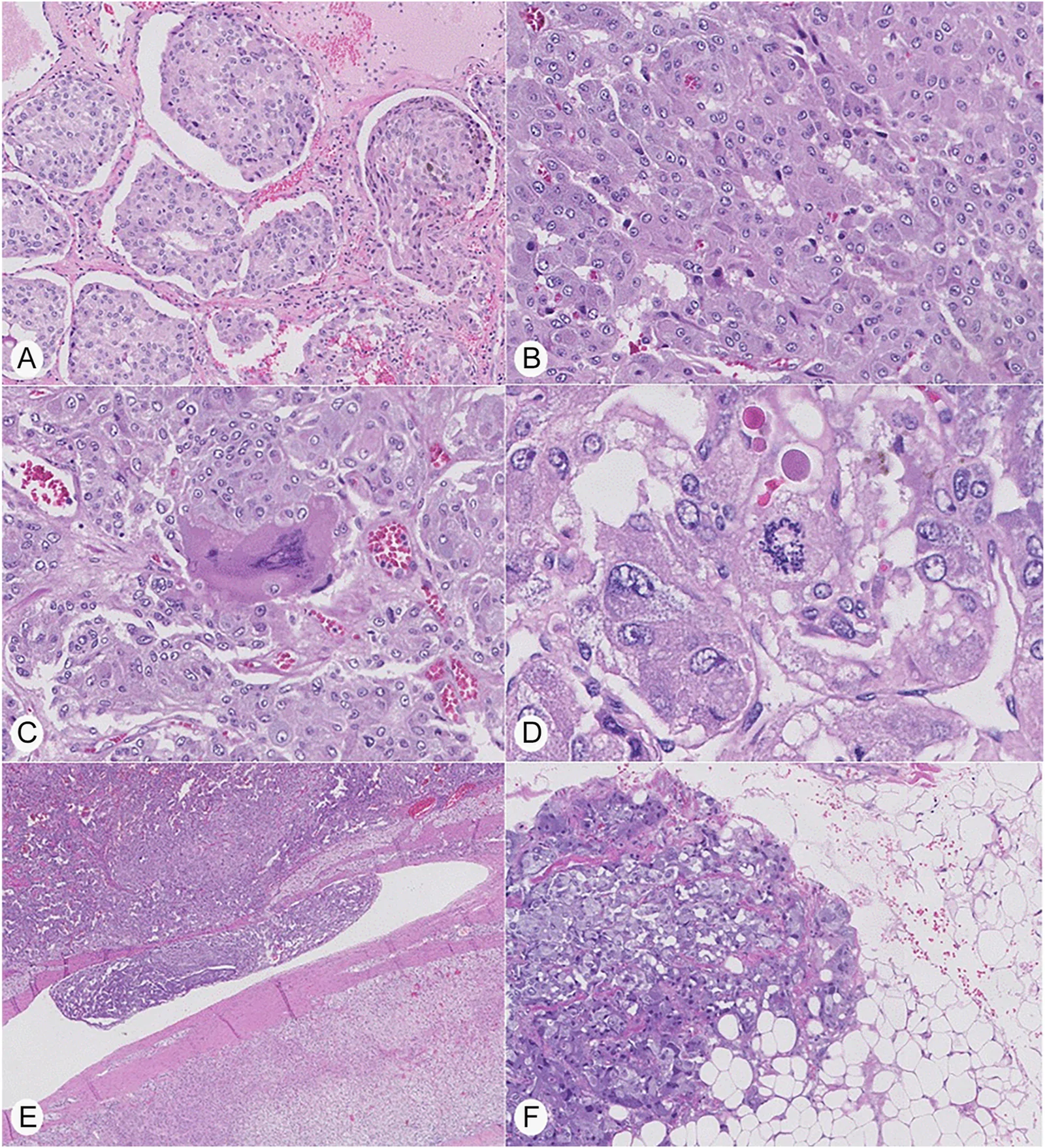
Histopathological features assessed for metastatic risk: (A) pheochromocytoma showing a well-differentiated nested (‘zellballen’) pattern. (B) Tumor with a less differentiated pattern and confluent nests. (C) Marked cellular pleomorphism. (D) Atypical mitosis. (E) Vascular invasion. (F) Periadrenal adipose tissue infiltration. (A, B, C, D, E, F) Hematoxylin–eosin stain.

Some of the histopathological features assessed for metastatic risk are shown in Fig. 2.

## Biochemical biomarkers

Biochemical biomarkers play a central role in the diagnosis, risk stratification, and follow-up of PPGL, providing functional insight into tumor differentiation status and metastatic potential (40).

### Plasma and urinary metanephrines

Measurement of plasma-free metanephrines (normetanephrine and metanephrine) and their urinary fractions remains the cornerstone of PPGL diagnosis and is strongly recommended by clinical guidelines (16, 41). Elevated levels of these metabolites have also been associated with metastatic disease, although findings are somewhat heterogeneous across studies (16, 42).

Biochemical phenotyping reflects tumor differentiation status (15, 42). Well-differentiated, typically adrenal tumors express phenylethanolamine N-methyltransferase (PNMT) and produce epinephrine, resulting in an adrenergic phenotype characterized by metanephrine secretion (15). In contrast, less differentiated tumors, more often extra-adrenal, lack PNMT expression and predominantly produce norepinephrine and dopamine, leading to a noradrenergic or dopaminergic phenotype, with elevated normetanephrine and 3-methoxytyramine levels as well as a higher likelihood of metastatic spread (14, 15, 16, 28, 42). A lower epinephrine-to-total catecholamine ratio has been associated with increased risk of recurrence and metastatic progression (42).

### Dopamine and 3-methoxytyramine

Dopamine production and its O-methylated metabolite, 3-methoxytyramine (3-MT), are among the most informative biochemical markers of metastatic risk and worse tumor prognosis in PPGL (15, 16, 28, 42, 43). These markers reflect poorly differentiated tumors, often associated with pseudohypoxic signaling and *SDHB*-related disease (15, 16, 28).

Elevated plasma 3-MT levels have consistently been linked to metastatic PPGL (15, 28, 42). Studies have shown that plasma 3-MT concentrations are approximately 4–5 times higher in patients with metastatic disease compared with those with localized tumors (15, 42). A cutoff of 0.16–0.28 nmol/L provides high specificity (85–95%) but moderate sensitivity (47–53%) for detecting metastasis (15, 28, 42).

Importantly, dopamine and norepinephrine-secreting tumors are associated with increased metastatic risk, supporting the concept of an ‘immature secretory phenotype’, linked to aggressive behavior (14, 15, 19, 28). Although 3-MT has limited sensitivity as a standalone biomarker, its predictive value improves when combined with clinical and genetic variables (28, 44).

### Chromogranin A

Chromogranin A (CgA) is a general neuroendocrine marker that can be elevated in PPGL and reflects tumor burden. However, its specificity is limited, as levels may be influenced by non-tumoral factors, such as renal insufficiency or proton pump inhibitor use, among others. Consequently, while CgA may be useful as an adjunct marker, particularly in non-secreting tumors, its role in predicting metastatic disease is less well established compared with catecholamine metabolites (40).

### Emerging circulating markers

Recent advances in metabolomics have identified circulating oncometabolites as promising biomarkers for PPGL risk stratification and disease monitoring (19, 24, 40, 44). In particular, succinate accumulation – characteristic of SDHx-deficient tumors – has emerged as a potential biomarker of tumor burden and metastatic progression (19, 24, 40, 44).

Elevated circulating succinate levels have been shown to correlate strongly with disease extent, particularly in *SDHB*-mutated metastatic PPGL, with reported correlations between serum succinate concentration and tumor load, and may allow discrimination between metastatic disease and assess disease burden (40). Moreover, longitudinal monitoring of succinate levels may provide a non-invasive tool for evaluating treatment response and detecting disease progression (40).

There is growing interest in developing liquid biopsy approaches for the detection of metabolites, such as succinate, fumarate, and alpha-ketoglutarate, in plasma (19, 24, 40, 44). Liquid chromatography/mass spectrometry methods allow sensitive and specific, fast, and inexpensive absolute quantification of serum metabolite levels (40). This technology is common in hospital laboratories, and development of such methods is quite simple, thus enabling the large-scale use of this test in clinical practice (40). Despite these advances, most emerging biomarkers remain investigational (40).

Additional metabolomic alterations, including changes in kynurenine pathway metabolites such as xanthurenic acid, have also been associated with poor prognosis and were considered an independent risk factor for metastatic disease, suggesting a broader role for metabolic profiling in PPGL (16).

Their integration with established biochemical, genetic, and clinical parameters – potentially supported by machine learning models – may significantly improve risk stratification and enable more personalized management strategies in patients with PPGL (28, 44).

## Circulating and liquid biopsy biomarkers

Beyond the established biochemical markers discussed above, liquid biopsy approaches are beginning to identify PPGL-derived molecular features in blood that may inform metastatic risk, prognosis, and disease monitoring. However, most studies remain constrained by retrospective design, limited cohort size, heterogeneity in clinical stage and genotype, non-standardized sampling, and insufficient external validation. Within these limitations, circulating microRNAs and circulating tumor DNA (ctDNA) are among the most promising analytes.

Drossart *et al.* analyzed 231 patients with PPGL, including 34 patients with metastatic disease, and reported that circulating miR-483-5p was the best-performing miRNA classifier of metastatic status, although with modest discrimination (AUC–ROC 0.64, 95% CI 0.52–0.77) (24). Notably, miR-483-5p levels correlated with tumor burden, and detection reached 100% in patients with liver metastases, suggesting a potential role in monitoring disease extent beyond simple metastatic status classification (24). This finding is biologically plausible, as miR-483-5p has also been implicated in tissue-based PPGL studies. A recent tissue study reported miR-483-5p upregulation in metastatic PPGL and in tumors with high Ki-67, supporting its potential role as a biomarker of aggressive biological behavior (45). This is consistent with earlier tissue-based profiling, which first identified miR-483-5p as overexpressed, alongside underexpression of the tumor-suppressor miRNAs, miR-15a and miR-16, in malignant compared with benign pheochromocytoma (46). A subsequent independent cohort confirmed miR-483-5p overexpression in malignant tumor tissue alongside miR-101 and miR-183, with diagnostic AUCs of 0.69–0.78. However, when these same three miRNAs were measured in patient serum, no difference was found between benign and malignant disease, illustrating that tissue-level discrimination does not always translate into a circulating biomarker (47). Since then, miR-101 has been reported as elevated specifically in serum in a separate cohort of SDHD-mutated malignant PPGL, with a diagnostic AUC of 0.79, in apparent contrast to the earlier negative serum finding (48). Serum miR-210 was inversely associated with malignant disease in a small pilot cohort, although this association did not reach significance on multivariable analysis, likely reflecting limited sample size (49). More recently, an integrative multi-omics analysis identified a six-miRNA prognostic signature in metastatic PPGL, in which miR-21-3p and miR-183-5p combined were the strongest predictors of time to progression. Here, miR-21-3p levels also correlated with mTOR pathway activation, suggesting potential utility as a predictive marker of mTOR inhibitor sensitivity (50). A competing endogenous RNA network analysis further identified hsa-miR-130b-3p and IREB2 as significantly overexpressed in metastatic compared with non-metastatic patients (51). Taken together, these findings support a biologically coherent, but still inconsistently reproduced, circulating miRNA landscape in PPGL, as reviewed elsewhere (52, 53).

Here, ctDNA represents another highly promising liquid biopsy modality in metastatic PPGL. A recent large cohort study of patients with metastatic PPGL reported that plasma ctDNA was clinically relevant for disease monitoring and prognostication, supporting its potential use as a non-invasive biomarker for longitudinal assessment and treatment stratification (54). Importantly, ctDNA may be particularly valuable in advanced disease where radiographic progression can be slow, heterogeneous, or difficult to interpret. However, before ctDNA can be incorporated into routine PPGL care, prospective studies must define optimal assay platforms, reporting metrics, sampling intervals, thresholds for molecular progression, and whether ctDNA-guided therapeutic decisions improve patient outcomes.

Overall, circulating biomarkers are among the most clinically advanced tools for PPGL risk assessment. Plasma metanephrines and 3-methoxytyramine already have established clinical relevance, whereas circulating miRNAs and ctDNA remain promising but investigational. Their future value will likely depend on integration with clinical features, genotype, imaging phenotype, tumor burden, and treatment context rather than use as isolated biomarkers.

## Imaging biomarkers

### Radiological imaging biomarkers

Radiological imaging contributes to risk stratification through the identification of features associated with malignancy, including tumor size, necrosis, and diffusion restriction, although no imaging characteristic can reliably predict or exclude metastatic behavior in isolation (43).

Diffusion-weighted magnetic resonance imaging (MRI) may offer a non-invasive means of assessing tumor cellularity (43), with restricted diffusion correlating with higher cellular density. Magnetic resonance spectroscopy (^1^H-MRS) enables the quantitative analysis of specific metabolites within tumor tissue (43). A ^1^H-MRS protocol optimized for succinate detection (SUCCES) has been developed to identify *SDHx* mutations noninvasively, supporting early diagnosis and aiding the confirmation of metastatic disease (43, 55). However, its clinical implementation requires an experienced team and considerable MRI machine time (55).

Radiomics is a quantitative imaging-based technique that enables the high-throughput extraction of a large number of tumor features from medical imaging studies (56, 57, 58). Many of these features are imperceptible to the human eye and are subsequently analyzed using computational methods, including machine learning algorithms and deep learning approaches, to identify patterns with potential diagnostic, prognostic, or predictive value (56, 57, 59). Radiomics has already been applied to predict tumor differentiation and perform risk stratification across several malignancies, including hepatocellular carcinoma, renal cell carcinoma, endometrial carcinoma, pancreatic neuroendocrine tumors, and gastrointestinal stromal tumors (56, 58). Recent studies have demonstrated that preoperative radiomic features of PPGLs correlate with the GAPP score (56), metastasis-free survival (57), and the distinction between benign and malignant tumors (60), suggesting a potential role for radiomics in the preoperative prediction of metastatic risk in PPGL (57, 58).

### Nuclear medicine imaging biomarkers

Several nuclear medicine imaging parameters have demonstrated utility as biomarkers of malignant potential in PPGL, particularly in the setting of metastatic disease. In a large prospective study, ^18^F-FDG PET/CT showed superior sensitivity for the detection of metastases compared with ^123^I-MIBG SPECT (80 vs 49%), with one-third of metastatic patients yielding false-negative MIBG scans; this advantage was most pronounced in SDHB/D-related disease (61). ^123^I-metaiodobenzylguanidine (MIBG) uptake was found to be significantly higher in malignant lesions (62).

A deep learning-based model applied to ^68^Ga-DOTATATE PET-CT has demonstrated reliable automated segmentation of metastatic PPGL lesions, enabling accurate quantification of tumor burden and potentially supporting objective monitoring of treatment response (43).

## Future directions

Recent clinical, biochemical, and molecular studies have substantially advanced our understanding of metastatic PPGL biology. Nevertheless, the available evidence remains limited by the rarity of the disease, retrospective study designs, fragmented datasets, heterogeneous definitions of progression and metastatic risk, and small numbers of metastatic events. These limitations continue to reduce statistical power, impair reproducibility, and delay clinical translation.

The development of reliable biomarkers for metastatic risk stratification, therapeutic target identification, and disease monitoring will require large, prospective, international registries. These efforts should incorporate standardized longitudinal collection of tumor tissue, plasma, germline and somatic genomic data, imaging, treatment exposure, response assessments, and survival outcomes. Harmonization of biospecimen processing, assay platforms, and clinical endpoints will be essential to distinguish biomarkers that are merely associated with aggressive disease from those that are analytically valid, clinically valid, and clinically useful.

From a therapeutic perspective, the field would benefit from closer alignment between translational research networks, academic consortia, patient registries, and pharmaceutical development programs (63). Such collaboration is critical to enable biomarker-driven trial design, rational combination strategies, and the evaluation of novel agents in molecularly defined PPGL subgroups. Given the rarity and biological heterogeneity of metastatic PPGL, future progress will likely depend on international collaboration, adaptive trial designs, and integrated biomarker programs embedded directly into clinical studies.

## Conclusion

The prediction of metastatic behavior in PPGL requires integration of multiple biomarker categories, as no single parameter is sufficient for accurate risk stratification. Germline *SDHB* mutation remains the strongest established predictor of metastatic disease, yet its incomplete penetrance highlights the importance of secondary molecular events, including ATRX loss, *TERT* activation, and elevated CDK1 expression, as critical modifiers of individual outcome. Histopathological scoring systems, biochemical phenotyping, and plasma 3-methoxytyramine provide complementary prognostic information in routine clinical practice.

Emerging biomarkers, encompassing genomic instability indices, tumor immune microenvironment characterization, and liquid biopsy approaches, are rapidly expanding the prognostic toolkit, although most remain investigational and require prospective validation. The key take-home messages for both clinicians and researchers are that dynamic, multiparametric risk stratification, integrating genetic, biochemical, histopathological, and molecular parameters, represents the most promising path toward accurate prognostication and personalized management of metastatic PPGL.

## Declaration of interest

The authors declare that there is no conflict of interest that could be perceived as prejudicing the impartiality of the work reported.

## Funding

This work received funding from PHEiPAS [Ayuda a la investigación de PHEiPAS, project: miR-15a y miR-16 como potenciales marcadores de enfermedad metastásica en pacientes con feocromocitoma o paraganglioma].

## Ethical approval

Ethical approval was not required for this study because it is a narrative review article based exclusively on the analysis and synthesis of previously published data. No new human participants were recruited; no identifiable personal data were collected; and no experimental procedures were performed. Therefore, approval by an institutional review board or ethics committee was not necessary in accordance with applicable institutional and national regulations.

## References

[1] Al Subhi AR, Boyle V & Elston MS. Systematic review: incidence of pheochromocytoma and paraganglioma over 70 years. J Endocr Soc 2022 6 bvac105. (10.1210/jendso/bvac105) 35919261 PMC9334688

[2] Berends AMA, Buitenwerf E, de Krijger RR, et al. Incidence of pheochromocytoma and sympathetic paraganglioma in the Netherlands: a nationwide study and systematic review. Eur J Intern Med 2018 51 68–73. (10.1016/j.ejim.2018.01.015) 29361475

[3] Burnichon N, Abermil N, Buffet A, et al. The genetics of paragangliomas. Eur Ann Otorhinolaryngol Head Neck Dis 2012 129 315–318. (10.1016/j.anorl.2012.04.007) 23078982

[4] Calissendorff J, Juhlin CC, Bancos I, et al. Pheochromocytomas and abdominal paragangliomas: a practical guidance. Cancers 2022 14 917. (10.3390/cancers14040917) 35205664 PMC8869962

[5] Fishbein L, Leshchiner I, Walter V, et al. Comprehensive molecular characterization of pheochromocytoma and paraganglioma. Cancer Cell 2017 31 181–193. (10.1016/j.ccell.2017.01.001) 28162975 PMC5643159

[6] Tischler AS, Kimura N & Mcnicol AM. Pathology of pheochromocytoma and extra-adrenal paraganglioma. Ann N Y Acad Sci 2006 1073 557–570. (10.1196/annals.1353.059) 17102124

[7] Lam AKY. Update on adrenal tumours in 2017 World Health Organization (WHO) of endocrine tumours. Endocr Pathol 2017 28 213–227. (10.1007/s12022-017-9484-5) 28477311

[8] Crona J, Taïeb D & Pacak K. New perspectives on pheochromocytoma and paraganglioma: toward a molecular classification. Endocr Rev 2017 38 489–515. (10.1210/er.2017-00062) 28938417 PMC5716829

[9] Snezhkina A, Pavlov V, Dmitriev A, et al. Potential biomarkers of metastasizing paragangliomas and pheochromocytomas. Life 2021 11 1179. (10.3390/life11111179) 34833055 PMC8619623

[10] Svendsen LK, Rasmussen ÅK, Klose MC, et al. Metastatic disease in phaeochromocytomas and paragangliomas in various genotypes-a systematic review and meta-analysis. J Clin Endocrinol Metab 2026 111 580–590. (10.1210/clinem/dgaf584) 41183483 PMC12819860

[11] Hamidi O, Young WF, Gruber L, et al. Outcomes of patients with metastatic phaeochromocytoma and paraganglioma: a systematic review and meta-analysis. Clin Endocrinol 2017 87 440–450. (10.1111/cen.13434) PMC5854189 28746746

[12] Juhlin CC. Challenges in paragangliomas and pheochromocytomas: from histology to molecular immunohistochemistry. Endocr Pathol 2021 32 228–244. (10.1007/s12022-021-09675-0) 33768452 PMC8116282

[13] Mete O & Juhlin CC. The evolving roles of pathologists in paragangliomas and pheochromocytomas. Virchows Arch Int J Pathol 2026 488 173–191. (10.1007/s00428-025-04379-w) 41432735

[14] Mete O, Asa SL, Gill AJ, et al. Overview of the 2022 WHO classification of paragangliomas and pheochromocytomas. Endocr Pathol 2022 33 90–114. (10.1007/s12022-022-09704-6) 35285002

[15] Alkaissi H, Taieb D, Lin FI, et al. Approach to the patient with metastatic pheochromocytoma and paraganglioma. J Clin Endocrinol Metab 2025 110 2946–2963. (10.1210/clinem/dgaf259) 40317194 PMC12448647

[16] Torresan F, Iacobone C, Giorgino F, et al. Genetic and molecular biomarkers in aggressive pheochromocytomas and paragangliomas. Int J Mol Sci 2024 25 7142. (10.3390/ijms25137142) 39000254 PMC11241596

[17] de Bresser CJM & de Krijger RR. The molecular classification of pheochromocytomas and paragangliomas: discovering the genomic and immune landscape of metastatic disease. Endocr Pathol 2024 35 279–292. (10.1007/s12022-024-09830-3) 39466488 PMC11659362

[18] Wachtel H & Nathanson KL. Molecular genetics of pheochromocytoma/paraganglioma. Curr Opin Endocr Metab Res 2024 36 100527. (10.1016/j.coemr.2024.100527) 39328362 PMC11424047

[19] Varghese J, Skefos CM & Jimenez C. Metastatic pheochromocytoma and paraganglioma: integrating tumor biology in clinical practice. Mol Cell Endocrinol 2024 592 112344. (10.1016/j.mce.2024.112344) 39182716

[20] Uher O, Hadrava Vanova K, Taïeb D, et al. The immune landscape of pheochromocytoma and paraganglioma: current advances and perspectives. Endocr Rev 2024 45 521–552. (10.1210/endrev/bnae005) 38377172 PMC11244254

[21] Calsina B, Piñeiro-Yáñez E, Martínez-Montes ÁM, et al. Genomic and immune landscape of metastatic pheochromocytoma and paraganglioma. Nat Commun 2023 14 1122. (10.1038/s41467-023-36769-6) 36854674 PMC9975198

[22] Thorsson V, Gibbs DL, Brown SD, et al. The Immune Landscape of Cancer. Immunity 2018 48 812–830.e14. (10.1016/j.immuni.2018.03.023) 29628290 PMC5982584

[23] Ghosal S, Hadrava Vanova K, Uher O, et al. Immune signature of pheochromocytoma and paraganglioma in context of neuroendocrine neoplasms associated with prognosis. Endocrine 2023 79 171–179. (10.1007/s12020-022-03218-1) 36370152 PMC10683554

[24] Drossart T, Buffet A, Janbain A, et al. Biomarkers improving genetic and metastatic disease prediction in paraganglioma: insights from a prospective study. J Clin Endocrinol Metab 2025 110 2193–2204. (10.1210/clinem/dgae797) 39541377

[25] Job S, Draskovic I, Burnichon N, et al. Telomerase activation and ATRX mutations are independent risk factors for metastatic pheochromocytoma and paraganglioma. Clin Cancer Res 2019 25 760–770. (10.1158/1078-0432.CCR-18-0139) 30301828

[26] Burnichon N, Cascon A, Schiavi F, et al. MAX mutations cause hereditary and sporadic pheochromocytoma and paraganglioma. Clin Cancer Res 2012 18 2828–2837. (10.1158/1078-0432.CCR-12-0160) 22452945

[27] Bausch B, Schiavi F, Ni Y, et al. Clinical characterization of the pheochromocytoma and paraganglioma susceptibility genes SDHA, TMEM127, MAX, and SDHAF2 for gene-informed prevention. JAMA Oncol 2017 3 1204–1212. (10.1001/jamaoncol.2017.0223) 28384794 PMC5824290

[28] Park SS, Ahn CH, Lee S, et al. Preoperative prediction of metastatic pheochromocytoma and paraganglioma using clinical, genetic, and biochemical markers: a cohort study. J Intern Med 2024 296 68–79. (10.1111/joim.13791) 38659304

[29] Astuti D, Morris M, Krona C, et al. Investigation of the role of SDHB inactivation in sporadic phaeochromocytoma and neuroblastoma. Br J Cancer 2004 91 1835–1841. (10.1038/sj.bjc.6602202) 15505628 PMC2410049

[30] Koh JM, Ahn SH, Kim H, et al. Validation of pathological grading systems for predicting metastatic potential in pheochromocytoma and paraganglioma. PLoS One 2017 12 e0187398. (10.1371/journal.pone.0187398) 29117221 PMC5678867

[31] Pierre C, Agopiantz M, Brunaud L, et al. COPPS, a composite score integrating pathological features, PS100 and SDHB losses, predicts the risk of metastasis and progression-free survival in pheochromocytomas/paragangliomas. Virchows Arch Int J Pathol 2019 474 721–734. (10.1007/s00428-019-02553-5) 30868297

[32] Zhuang Z, Yang C, Lorenzo F, et al. Somatic HIF2A gain-of-function mutations in paraganglioma with polycythemia. N Engl J Med 2012 367 922–930. (10.1056/NEJMoa1205119) 22931260 PMC3432945

[33] Buffet A, Smati S, Mansuy L, et al. Mosaicism in HIF2A-related polycythemia-paraganglioma syndrome. J Clin Endocrinol Metab 2014 99 E369–E373. (10.1210/jc.2013-2600) 24276449

[34] Thompson LDR. Pheochromocytoma of the adrenal gland scaled score (PASS) to separate benign from malignant neoplasms: a clinicopathologic and immunophenotypic study of 100 cases. Am J Surg Pathol 2002 26 551–566. (10.1097/00000478-200205000-00002) 11979086

[35] Kimura N, Takayanagi R, Takizawa N, et al. Pathological grading for predicting metastasis in phaeochromocytoma and paraganglioma. Endocr Relat Cancer 2014 21 405–414. (10.1530/ERC-13-0494) 24521857

[36] Wachtel H, Hutchens T, Baraban E, et al. Predicting metastatic potential in pheochromocytoma and paraganglioma: a comparison of PASS and GAPP scoring systems. J Clin Endocrinol Metab 2020 105 e4661–e4670. (10.1210/clinem/dgaa608) 32877928 PMC7553245

[37] Li Q, Lan Z, Jiang Y, et al. Validation and evaluation of 5 scoring systems for predicting metastatic risk in pheochromocytoma and paraganglioma. Am J Surg Pathol 2024 48 855–865. (10.1097/PAS.0000000000002238) 38712603

[38] Thompson LDR, Gill AJ, Asa SL, et al. Data set for the reporting of pheochromocytoma and paraganglioma: explanations and recommendations of the guidelines from the international collaboration on cancer reporting. Hum Pathol 2021 110 83–97. (10.1016/j.humpath.2020.04.012) 32407815 PMC7655677

[39] van Nederveen FH, Gaal J, Favier J, et al. An immunohistochemical procedure to detect patients with paraganglioma and phaeochromocytoma with germline SDHB, SDHC, or SDHD gene mutations: a retrospective and prospective analysis. Lancet Oncol 2009 10 764–771. (10.1016/S1470-2045(09)70164-0) 19576851 PMC4718191

[40] Lamy C, Tissot H, Faron M, et al. Succinate: a serum biomarker of SDHB-mutated paragangliomas and pheochromocytomas. J Clin Endocrinol Metab 2022 107 2801–2810. (10.1210/clinem/dgac474) 35948272

[41] Plouin PF, Amar L, Dekkers OM, et al. European Society of Endocrinology Clinical Practice Guideline for long-term follow-up of patients operated on for a phaeochromocytoma or a paraganglioma. Eur J Endocrinol 2016 174 G1–G10. (10.1530/EJE-16-0033) 27048283

[42] Schreiner F & Beuschlein F. Disease monitoring of patients with pheochromocytoma or paraganglioma by biomarkers and imaging studies. Best Pract Res Clin Endocrinol Metab 2020 34 101347. (10.1016/j.beem.2019.101347) 31662271

[43] Choucair A, Zdunek A, Liao M, et al. Precision imaging and evolving therapies in paragangliomas and pheochromocytomas: from molecular diagnostics to imaging-guided management. Insights Imaging 2026 17 37. (10.1186/s13244-025-02195-z) 41661399 PMC12886687

[44] Juhlin CC & Mete O. Hot trends in pheochromocytoma and paraganglioma: are we getting closer to personalized dynamic prognostication? Turk Patoloji Derg 2024 40 143–148. (10.5146/tjpath.2024.13681) 39171856 PMC11402475

[45] Thanasoula F, Yavropoulou MP, Kyriakopoulos G, et al. Clinicopathological data and the role of miRNA expression in patients with pheochromocytomas/paragangliomas. Front Endocrinol 2025 16 1716806. (10.3389/fendo.2025.1716806) PMC12711539 41427045

[46] Meyer-Rochow GY, Jackson NE, Conaglen JV, et al. MicroRNA profiling of benign and malignant pheochromocytomas identifies novel diagnostic and therapeutic targets. Endocr Relat Cancer 2010 17 835–846. (10.1677/ERC-10-0142) 20621999

[47] Patterson E, Webb R, Weisbrod A, et al. The microRNA expression changes associated with malignancy and SDHB mutation in pheochromocytoma. Endocr Relat Cancer 2012 19 157–166. (10.1530/ERC-11-0308) 22241719 PMC4716660

[48] Zong L, Meng L & Shi R. Role of miR-101 in pheochromocytoma patients with SDHD mutation. Int J Clin Exp Pathol 2015 8 1545–1554. PubMed PMID: 25973039; PubMed Central PMCID: PMC4396236. 25973039 PMC4396236

[49] Ruff SM, Ayabe RI, Malekzadeh P, et al. MicroRNA-210 may be a preoperative biomarker of malignant pheochromocytomas and paragangliomas. J Surg Res 2019 243 1–7. (10.1016/j.jss.2019.04.086) 31146085 PMC7478339

[50] Calsina B, Castro-Vega LJ, Torres-Perez R, et al. Integrative multi-omics analysis identifies a prognostic miRNA signature and a targetable miR-21-3p/TSC2/mTOR axis in metastatic pheochromocytoma/paraganglioma. Theranostics 2019 9 4946–4958. (10.7150/thno.35458) 31410193 PMC6691382

[51] Wang Z, Li Y, Zhong Y, et al. Comprehensive analysis of aberrantly expressed competitive endogenous RNA network and identification of prognostic biomarkers in pheochromocytoma and paraganglioma. Onco Targets Ther 2020 13 11377–11395. (10.2147/OTT.S271417) 33192072 PMC7654541

[52] Tsoli M, Daskalakis K, Kassi E, et al. A critical appraisal of contemporary and novel biomarkers in pheochromocytomas and adrenocortical tumors. Biology 2021 10 580. (10.3390/biology10070580) 34201922 PMC8301201

[53] Igaz P, Igaz I, Nagy Z, et al. MicroRNAs in adrenal tumors: relevance for pathogenesis, diagnosis, and therapy. Cell Mol Life Sci 2015 72 417–428. (10.1007/s00018-014-1752-7) 25297921 PMC11114066

[54] Toledo RDA, Arenillas C, Kim E, et al. 37O Plasma circulating tumor DNA (ctDNA) as a clinically relevant biomarker for disease monitoring and prognostication in metastatic pheochromocytoma and paraganglioma (mPPGL). ESMO Open 2025 10. (10.1016/j.esmoop.2025.104348)

[55] Lussey-Lepoutre C, Bellucci A, Morin A, et al. In vivo detection of succinate by magnetic resonance spectroscopy as a hallmark of SDHx mutations in paraganglioma. Clin Cancer Res 2016 22 1120–1129. (10.1158/1078-0432.CCR-15-1576) 26490314

[56] Zhou Y, Zhan Y, Zhao J, et al. CT-based radiomics deep learning signatures for non-invasive prediction of metastatic potential in pheochromocytoma and paraganglioma: a multicohort study. Insights Imaging 2025 16 81. (10.1186/s13244-025-01952-4) 40185919 PMC11971077

[57] Zhou Y, Zhan Y, Zhao J, et al. CT-Based radiomics analysis of different machine learning models for discriminating the risk stratification of pheochromocytoma and paraganglioma: a multicenter study. Acad Radiol 2024 31 2859–2871. (10.1016/j.acra.2024.01.008) 38302388

[58] Zhao J, Zhan Y, Zhou Y, et al. CT-based radiomics research for discriminating the risk stratification of pheochromocytoma using different machine learning models: a multi-center study. Abdom Radiol 2024 49 1569–1583. (10.1007/s00261-024-04279-8) 38587628

[59] Crimì F, Agostini E, Toniolo A, et al. CT texture analysis of adrenal pheochromocytomas: a pilot study. Curr Oncol 2023 30 2169–2177. (10.3390/curroncol30020167) 36826128 PMC9955696

[60] Laderian B, Ahmed FS, Zhao B, et al. Role of radiomics to differentiate benign from malignant pheochromocytomas and paragangliomas on contrast enhanced CT scans. J Clin Oncol 2019 37 (Supplement 15) e14596. (10.1200/JCO.2019.37.15_suppl.e14596)

[61] Timmers HJ, Chen CC, Carrasquillo JA, et al. Staging and functional characterization of pheochromocytoma and paraganglioma by 18F-fluorodeoxyglucose (18F-FDG) positron emission tomography. J Natl Cancer Inst 2012 104 700–708. (10.1093/jnci/djs188) 22517990 PMC3341309

[62] Maurea S, Cuocolo A, Reynolds JC, et al. Diagnostic imaging in patients with paragangliomas. Computed tomography, magnetic resonance and MIBG scintigraphy comparison. Q J Nucl Med 1996 40 365–371. 9050342

[63] Arenillas C & Toledo RA. FDA fast-track approval of belzutifan is a milestone in rare cancer therapy. Nat Rev Endocrinol 2025 21 739–740. (10.1038/s41574-025-01183-z) 40935878

